# Thoracoabdominal Computed Tomography in Trauma Patients: A Cost-Consequences Analysis

**DOI:** 10.5812/traumamon.19219

**Published:** 2014-08-01

**Authors:** Raoul van Vugt, Digna R. Kool, Monique Brink, Helena M. Dekker, Jaap Deunk, Michael J. Edwards

**Affiliations:** 1 Department of Surgery, Radboud University Medical Center, Nijmegen, the Netherlands; 2 Department of Radiology, Radboud University Medical Center, Nijmegen, the Netherlands

**Keywords:** Costs and Cost Analysis, Wounds and Injuries, Tomography, X-Ray Computed, Thorax, Abdomen

## Abstract

**Background:**

CT is increasingly used during the initial evaluation of blunt trauma patients. In this era of increasing cost-awareness, the pros and cons of CT have to be assessed.

**Objectives:**

This study was performed to evaluate cost-consequences of different diagnostic algorithms that use thoracoabdominal CT in primary evaluation of adult patients with high-energy blunt trauma.

**Materials and Methods:**

We compared three different algorithms in which CT was applied as an immediate diagnostic tool (rush CT), a diagnostic tool after limited conventional work-up (routine CT), and a selective tool (selective CT). Probabilities of detecting and missing clinically relevant injuries were retrospectively derived. We collected data on radiation exposure and performed a micro-cost analysis on a reference case-based approach.

**Results:**

Both rush and routine CT detected all thoracoabdominal injuries in 99.1% of the patients during primary evaluation (n = 1040). Selective CT missed one or more diagnoses in 11% of the patients in which a change of treatment was necessary in 4.8%. Rush CT algorithm costed € 2676 (US$ 3660) per patient with a mean radiation dose of 26.40 mSv per patient. Routine CT costed € 2815 (US$ 3850) and resulted in the same radiation exposure. Selective CT resulted in less radiation dose (23.23 mSv) and costed € 2771 (US$ 3790).

**Conclusions:**

Rush CT seems to result in the least costs and is comparable in terms of radiation dose exposure and diagnostic certainty with routine CT after a limited conventional work-up. However, selective CT results in less radiation dose exposure but a slightly higher cost and less certainty.

## 1. Background

In trauma care, it is imperative to detect potentially life-threatening injuries as quickly and effectively as possible. Outcomes in terms of morbidity and mortality seem to improve if a uniform, standard protocol of rapid evaluation and treatment of trauma patients is used (1-4). For this reason, in many centers the Advanced Trauma Life Support (ATLS) principles are advocated for initial evaluation. ATLS advises the use of conventional radiography (CR), focused abdominal sonography in trauma (FAST), and computed tomography (CT) depending on the patient’s status. CT in trauma has been shown to be superior to CR and FAST in detecting and excluding traumatic injuries (5). Moreover, CT may have an additional effect on treatment strategy as well (6). However, drawbacks of CT are exposure to ionizing radiation (7), costs, possibility of unnecessary medical management, loss of time, and delay in treatment (8).

Due to its diagnostic advantages, thoracoabdominal CT (TACT) is increasingly employed in hospital protocols for the initial evaluation of patients with blunt trauma. However, in the era of increasing cost awareness, it is necessary to weigh the pros and cons of CT in a financial perspective as well. To our knowledge, no study concerning the cost-effectiveness of different diagnostic strategies using TACT in blunt trauma has been done.

## 2. Objectives 

The purpose of this study was to evaluate the relevant costs and diagnostic benefits of three different CT imaging algorithms in the initial evaluation of thoracoabdominal injuries in patients with high-energy blunt trauma.

## 3. Materials and Methods

### 3.1. Diagnostic Algorithms

**Figure 1.**
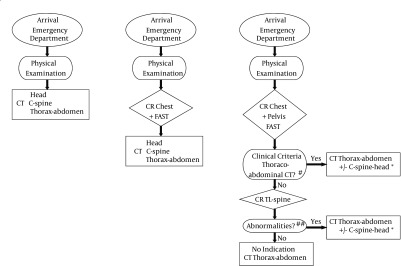
Three Different Algorithms Utilizing Computed Tomography Used in This Study to Diagnose Thoracoabdominal Injuries * Patients with one or more criteria for head CT (Appendix A); Patients with positive NEXUS criteria, those who met Canadian C-spine rules, or patients in whom criteria for CT of other regions were met (10, 11); #, clinical criteria for thoracoabdominal CT (Appendix A); ##, suspicion for a fracture or spinal malalignment; TL-spine, thoracolumbar spine; C-spine, cervical spine.

Based on a reference case-based approach of previous studies and recent literature (1, 6, 9-11), we developed three hypothetic algorithms for radiologic evaluation of patients with blunt trauma (Figure 1). Algorithms that were investigated included two low-threshold algorithms, in which TA CT was obtained in all patients, and one algorithm with higher threshold for imaging with a selective CT. 

In the first algorithm, all patients underwent TACT immediately after primary evaluation and stabilization without prior CXR/FAST (rush CT). In the second algorithm, all patients underwent TACT after limited conventional work-up consisting of chest and/or pelvic XR and FAST (routine CT). In the third algorithm, patients underwent TACT only if one or more criteria for chest CT and/or abdominal CT were met (selective CT). In this final algorithm, thoracic and/or lumbar spine XR were performed only when none of the other criteria for the specific CT were met (Appendix A) (10, 11). 

Head and cervical spine CT were not considered in this analysis. We built a strategic decision tree by using TreeAge 2009 Suite software (TreeAge Software Inc., Williamstown, MA, USA) to investigate as well as to compare the different diagnostic algorithms.

### 3.2. Study Sample and Setting

In this prospective cohort study, three different algorithms were tested on the data collected from 1040 consecutive adult patients with blunt trauma at a level one trauma center (clinical trial registration No. NCT00228111, http://www.clinicaltrials.gov/; Appendix B). All patients underwent physical examination according to ATLS, laboratory investigations, chest, pelvic, and complete spine XR, FAST, and cervical spine, chest, abdomen, and pelvic CT. Head CT was performed according to its indications (Appendix A) (12). During the study period, a 16-channel multidetector row CT with automated tube current modulation (Siemens Medical Systems, Erlangen, Germany) was used. Diagnostic protocols of radiologic investigations are provided in Appendix C. Based on interpretations of XR, FAST, and CT, the trauma team started or changed patient management as needed. Follow-up period was six months. All charts were re-reviewed to establish whether or not initially missed injuries had manifested over time. The data were recorded and entered in a customized database.

### 3.3. Outcomes

Primary outcome of the present study assessed the financial costs from a hospital perspective for each algorithm during initial patient evaluation and diagnostic work-up at our emergency ward. Another outcome measure evaluated the ionizing radiation exposure in each algorithm. These outcomes were compared with the previously published diagnostic value of each algorithm (6, 9).

### 3.4. Financial Costs

We calculated financial costs of the emergency department from a hospital perspective by using a micro-cost approach according to Dutch guidelines for economic research in healthcare (13). We collected information on financial costs during a time horizon including primary evaluation and diagnostic work-up of a trauma patient at the emergency department. This included information about staff, material, equipment, supporting departments, and overhead. Integral costs of these resources were calculated as product of the volume of resources per patient and their unit costs. We thereafter calculated incremental costs for each algorithm. All costs were reported in Euros (€) and US dollar (US$) for the year 2011. If no information on costs that year was available, costs were obtained from previous years and corrected for inflation by using the Dutch consumer health index (available at: http://statline.cbs.nl).

### 3.5. Unit Resources and Time Durations

For cost calculations on staff and facility space occupation, we used prospective time measurements performed by an investigator who was not involved in patient care.

### 3.6. Personnel Units

Depending on the severity of injuries, different staff combinations were needed per evaluation and diagnostic work-up. We assumed that two nurses, one radiographer, one anesthesiology technician, one resident of surgery, emergency medicine, neurology, radiology, and anesthesiology were occupied until complete evaluation and diagnostic work-up was complete. We furthermore assumed that one trauma surgeon and a radiologist were occupied no longer than half of the complete work-up time. 

### 3.7. Medical Supplies

Because patient-specific adjuncts such as supplies for intubation, chest drainage, stomach drainage, and pelvic stabilization were used in only a subgroup of patients included in our analysis, we obtained the frequency of using these devices from our customized database.

### 3.8. Unit Costs

Staff costs per hour were calculated according to the Dutch economic analysis guidelines (13). Our calculations for the costs of supervising staff, residents, nurses, radiographers, and anesthesiology technicians were based on the employee costs. Costs were based on wages at university medical centers in the Netherlands during 2011.

Equipment costs included costs of CXR including radiography system, analog-to-digital converter, digital working stations, sonography machine, and CT scanner. Calculating costs of the radiographic imaging were based on purchase price and value-added tax (VAT), which were adjusted to price index for healthcare. This income also calculated depreciation and interest costs per year. Costs of intravenous contrast material in general and the patient-specific adjuncts were assessed along with costs of laboratory diagnostic tests (2003; available at http://www.cvz.nl) corrected for inflation by using the Dutch consumer health index.

Facility space costs included trauma room and CT suite. Overhead costs were calculated as 35% of personnel and supplies costs.

### 3.9. Radiation Exposure

We calculated the radiation exposure of each XR investigations algorithm (Appendix C). It was performed by a phantom study for three representative patient configurations, followed by a calculation to access the effective radiation dose (see Appendix D).

Effective radiation doses (mSv) of chest, abdominal, and thoracoabdominal CT were calculated using different protocols and a random sample of 200 patients. We calculated the radiation dose for these patients and subsequently established a mean dose per patient for each algorithm (Appendix D).

## 4. Results

### 4.1. Demographic Data

Amongst 1040 patients, 729 (70%) were males. The mean age of the participants was 37 ± 18 years, median injury severity score (ISS) was 14. Mortality rate after six months was 5.5%. Among the study patients, 589 (57%) had injuries on TACT; 502 (48%) and 309 (30%) patients had chest and abdominal injuries, respectively. In 99.1% of patients, all injuries were initially detected by TACT. In nine patients, 12 injuries that were initially missed by TACT were detected during laparotomy due to other indications or during follow-up; these injuries included bowel perforation (4) and injuries to the liver (2), pancreas (2), spleen (2), bladder (1), and diaphragm (1). During initial evaluation, all the patients were ventilatory and hemodynamically stable or responded well to primary resuscitation (e.g., fluid therapy, endotracheal intubation, and chest-tube placement); otherwise, patients were excluded from the study. All patients underwent both conventional work-up and CT.

### 4.2. Diagnostic Value

According to the algorithms, all patients in both rush and routine CT algorithms underwent TACT. The injuries were detected in 99.1% of these patients. In the selective CT algorithm, 903 patients fulfilled criteria for TACT and immediately underwent CT without previous thoracolumbar XR. The remaining 137 patients underwent thoracolumbar XR (n = 116) and lumbar XR (n = 21). In this algorithm, 108 patients did not undergo TACT due to the absence of an indication according to the protocol in Appendix A. 

Missed injuries in the selective CT algorithm were predominantly free fluid and Organ Injury Scale (14) I-II injuries of the spleen, kidney, liver, adrenal injuries, small acetabular fractures, stable vertebral body fractures and transverse process fractures, pneumothorax, pulmonary contusions, fractures of rib, scapula, and sternum. 

Based on the CXR, 32 patients received chest tube drainage. Performing FAST did not directly result in any acute interventions although the indications for laparotomy were already made in several cases before CT. Pelvic CR resulted in an intervention in 17 patients. 

The time for physical evaluation in 57 patients was 19 minutes (mean, 21; range, 7-47); time for evaluation including XR and sonography was 21 minutes (mean, 23; range, 9-47); time for total work-up including head and cervical spine CT was 77 minutes (mean, 85; range, 62-138).

### 4.3. Costs

The calculated costs for supervising staff, residents, and nurses/radiographers/anesthesiology technicians were € 106 (US$ 145), € 40, (US$ 55) and € 33 (US$ 45) per hour, respectively. The established equipment cost prices were € 36 (US$ 49) , € 41 (US$ 56) , and € 4 (US$ 6) per CT, FAST, and CXR, respectively. 

Costs for disposables (€ 215 (US$ 294)) and laboratory investigations (€ 84 (US$ 115)) were the same in all three algorithms. Variable costs consisted of costs for diagnostic equipment, staff, housing, and overhead costs. Total cost calculated from a hospital perspective for each algorithm were € 2743 (US$ 3752), € 2945 (US$ 4029), and € 2890 (US$ 3954) for the rush, routine, and selective CT algorithms, respectively (Table 1). The staff costs constituted the largest part of the total cost.

**Table 1.** Financial Cost Estimates and Probabilities per Patient ^a,b^

	Rush CT Algorithm	Routine CT Algorithm	Selective CT Algorithm
**Disposables**	215	215	215
**Laboratory Investigations**	84	84	84
**Staff**	1422	1557	1498
**Housing**	352	309	290
**Overhead**	620	683	713
**Diagnostic Equipment (FAST, CR, and CT) and Contrast Drugs**	50	96	90
**Total**	2743	2945	2890

^a^Costs were derived from a micro-cost analysis and are represented in Euros (2011).

^b^Abbreviations: CR, conventional radiography; CT, computed tomography; and FAST, focused abdominal sonography in trauma.

### 4.4. Radiation

The calculated radiation dose of a 74 kilogram patient was 0.026 mSv for an anteroposterior chest XR, 0.26 mSv for an anteroposterior pelvic XR, 0.153 mSv for anteroposterior and lateral thoracic spine XR, and 0.515 mSv for anteroposterior and lateral lumbar XR. The effective dose estimates for either chest or abdomen CT were 8.81 and 12.85 mSv, respectively; the dose for thoracoabdominal CT was 19.5 mSv. Patients with a lower weight received a lower radiation dose, patients with a higher weight received a higher radiation dose (Table 2).

**Table 2.** Radiation Dose Estimates^a^

	Radiation Dose, mSv		
	Minimum, 45 kg	Maximum, 100 kg	Mean, 74 kg
**Chest XR, AP**	0.022	0.035	0.026
**Pelvic XR, AP**	0.369	0.574	0.260
**Thoracic Spine XR, AP & Lat**	0.210	0.301	0.153
**Lumbar Spine XR, AP & Lat**	0.398	0.623	0.515
**Brain CT **	1.50	3.00	2.00
**Cervical Spine CT **	2.20	6.00	3.00
**Chest CT**	5.67	16.03	8.81
**Abdominal CT **	7.95	22.50	12.85
**Thoracoabdominal CT **	12.56	35.55	19.5

^a^Abbreviations: AP, anterior-posterior; XR, radiography; CT, computed tomography; Lat, lateral.

### 4.5. Costs and Radiation

After performing strategic decision tree analysis (Table 3), the mean costs per patient for the rush algorithm (€ 2676 / US$ 3660) was the lowest, followed by the selective CT algorithm that costed € 95 (US$ 130). The most expensive algorithm was the routine CT algorithm with a cost of € 139 (US$ 190) per patient Mean radiation dosage per patient was significantly lower in the selective CT algorithm with 23.23 mSv. The rush and routine CT algorithm resulted in a mean radiation dose of 26.40 and 26.69 mSv, respectively.

**Table 3.** Financial Costs and Radiation Exposure of Three Different Diagnostic Strategies Employing Thoracoabdominal Computed Tomography in Adult Patients with Blunt Trauma ^a^

Algorithm	Rush CT	Routine CT	Selective CT
**Costs per Patient, €**	2676	2815	2771
**Incremental Costs per Patient, €**	NA	139	95
**Mean Radiation per Patient, mSv**	26.40	26.69	23.23

^a^For definitions of algorithms, see Figure 1; NA, not applicable (reference group for incremental cost calculation); CT, computed tomography.

## 5. Discussion

We evaluated the costs of three different algorithms that used CT for evaluating thoracoabdominal injuries after high-energy blunt trauma. The most important parts of the total costs were time-and staff-related. The sole use of diagnostic tools was not that expensive. This explains why the rush CT algorithm was cheaper. In this algorithm, total diagnostic work-up took the least time and consequently was less staff occupying. Selective and routine CT algorithms took more time and consequently, were more expensive.

Rush and routine CT algorithms had the same radiation and diagnostic value per patient, but costs were in favor of the rush CT algorithm. In this regard, rush CT seems to have the financial advantage over routine CT. Routine CT is a simple and clear algorithm, with a short work-up with CXR and FAST used to exclude or treat serious problems; moreover, it can be used safely in less stable patients. In the rush CT algorithm, less stable patients are potentially at risk because they go straight to the CT room and CT is not always immediately available. Moreover, performing acute interventions in the CT room is potentially more difficult than in the trauma room, because the CT room is usually smaller, has basic equipment, and a different climate control (focused on best practice for CT scanner). In our study, 32 patients received chest tube drainage due to findings on CXR and 17 patients underwent an intervention based on pelvic XR. Perhaps it is preferable to exclude time-consuming diagnosis with CR and to stabilize patients before transfer to the CT room. Compared to rush CT, routine CT algorithm is relatively slow and thus more expensive. In contrast to the selective CT algorithm, the rush CT is simpler; moreover, it is the fastest algorithm this makes it the cheapest diagnostic algorithm. On the other hand, less CT scans are performed in the selective CT algorithm that reduces patient irradiation (3 mSv) and the number of patients transferred to the CT room. However, this algorithm is somewhat cumbersome; it takes more time to conduct this makes it more expensive; and, it has more missed injuries.

There is still an ongoing controversy concerning whether CT should be performed routinely or be preserved for selective situations (5, 15, 16). Costs, time, and radiation exposure have to be taken into account to make a choice. We think that until the rush CT algorithm is proven to be safe (15), selective CT is preferred due to radiation reduction and the least unnecessary CT imaging; extra costs are limited (16).

This cost-consequences analysis has its own limitations. First, the cost-consequences of the three different algorithms were retrospectively determined in the same population (10, 11). This study was an empiric/reference case-based cost-consequences analysis. However, no sensitivity analysis was performed to show how the results depended on the assumptions made (17). It is difficult to extrapolate the findings to different countries due to demographic, epidemiologic and cultural factors, system of healthcare and its availability, differences in medical treatment, financing of healthcare, and absolute and relative price indexes (18). The CT in our hospital was located in the emergency department; however, when it was not employed for trauma-related purposes, it was used for other acute and regular assessments. This resulted in a high frequency of usage, which would expedite the depreciation of CT scanner. Therefore, caution in extrapolating these findings is needed. Finally, it would have been preferable if the improvement in quality of life had been used as an outcome measure in this study. Alternatively, it would have been helpful if the financial consequences of missed injuries could be taken into account.

In conclusion, we can state that the majority of costs for the evaluation of trauma patients in particular were personnel costs. Costs for the radiologic examinations themselves were only a minor part. The investigated three algorithms were close in terms of costs and radiation. The rush CT algorithm was the fastest and consequently, the cheapest diagnostic algorithm and comparable in terms of radiation exposure and diagnostics certainty with routine CT. However, selective CT resulted in less radiation, slightly higher cost and more injuries missed.

## Appendix A

**Table:** Indications of Selective Computed Tomography after High-Energy Blunt Trauma ^a^

Criteria
**Criteria for Head CT**
Presence of One of The Following Major Criteria
Pedestrian or bicycle versus motor vehicle
Ejection from vehicle
Vomiting
Posttraumatic amnesia of 1) > 4 hours and/or 2) at time of presentation at the ED
Clinical signals of a skull fracture
GCS ≤ 14 at time of presentation at the ED
Decline of at least 2 points in GCS one hour after presentation at the ED
Usage of anticoagulant drugs (coumarin derivatives) or a coagulation Disorder
Posttraumatic insult
Focal neurological deficit
Presence of Two or More of The Following Minor Criteria
Fall from > 3 m height
Persistent anterograde amnesia
Posttraumatic amnesia of 2-4 hours
Superficial head injuries (excluding the face)
Loss of consciousness
Decline of 1 point in GCS one hour after presentation
Age > 40 years
**Criteria for Cervical Spine CT **
Presence of One of the Following Major Criteria
Pain in cervical midline
Focal neurological deficit
Painful distracting injury
Intoxication
Decreased consciousness
**Criteria For Chest/Abdominal CT **
Presence of One of The Following Major Criteria
Clinical Criteria
Age ≥ 55 years
Hypotension (systolic blood pressure < 90 mm Hg)
GCS ≤ 14, tracheal intubation, sedation or intoxication
Abnormal finding during physical examination of the chest (diminished breath sounds, subcutaneous emphysema, pain under pressure, or extensive hematomas or lacerations on the chest)
Abnormal finding during physical examination of the abdomen (pain under pressure, distention, abdominal guarding, or extensive hematomas of lacerations on the abdomen)
Abnormal Finding During Physical Examination of The Thoracolumbar Spine (Pain on Palpation of The Spine, Focal Neurological Deficit, Extensive Hematomas of Lacerations on The Back)
Clinical Suspicion of a Pelvic Fracture
Macroscopically hematuria
Clinical suspicion of a long bone fracture (femur, tibia, fibula, humerus, radius and/or ulna)
Base excess < 3 mmol/L
Hemoglobin < 6 mmol/L
Radiological Criteria
Suspected Injuries on CXR
Lung contusion, hemothorax, pneumothorax, subcutaneous emphysema, abnormal mediastinum suggestive for a mediastinal hematoma, suspicion for diaphragmatic injury, rib fracture or a fracture of the spine, scapula and/or clavicle
Abnormalities on the Pelvic XR or FAST
Suspicion injury on CR of the pelvis (fracture of the pelvis or femur, sacroiliac luxation, symphysiolysis or a luxation of the hip joint)
Abnormalities on FAST
Presence of free fluid, abnormal organs, or pericardial fluid
Abnormalities on Thoracolumbar spine XR
Suspicion of a fracture or spinal malalignment

^a^ abbreviations: GCS, Glasgow Coma Scale; ED, emergency department; CT, computed tomography; CXR, chest radiography; and FAST, focused abdominal sonography in trauma.

## Appendix B

**Table:** Inclusion Criteria for Adult (> 16 Years Old) Patients With High-Energy Blunt Trauma Protocol Between June 2005 and August 2008 (11) ^a^

	Definitions
**Inclusion Criteria**	
Vital Problems	
Airway patency	As declared by anesthesiologist
Breathing problems	Respiratory rate ≥ 30/min
Circulatory problems	Heart rate ≥ 120/min; Systolic blood pressure < 100 mm Hg; Capillary refill > 4 s; External blood loss > 500 mL
Neurologic problems	GCS ≤ 13
Physical examination	
Clinically evident fractures ≥ 2 long bones	As declared by attending surgeon
Clinically evident pelvic ring fracture	As declared by attending surgeon
Signs of unstable vertebral fractures or spinal cord compression	As declared by attending surgeon
Mechanism of injury	
High-energy mechanism of injury as declared by prehospital emergency medical service	Fall from > 3 m height; motor vehicle accident with the speed of ≥ 50 km/h, ejection from a vehicle; car rollover; cabin shortening ≥ 50 cm; hit by (motor) cyclist ≥ 30 km/h
High-energy crush injury to torso	Pedestrian vs. motor vehicle ≥ 10 km/h; squeezed underneath or between heavy objects
**Exclusion criteria**	
CT not feasible/appropriate	
Shock class IIIB/IV	Pulse rate ≥ 120/min or systolic blood pressure < 100 mm Hg and nonresponsive to volume therapy
Immediate neurosurgical intervention	As declared by neurosurgeon
Pregnancy	Suspicious by history or AUS
Dead on arrival	As declared by attending surgeon

^a^ Abbreviations: GCS, Glasgow Coma Scale; CT, computed tomography; AUS, abdominal ultrasonography.

## Appendix C

**Table:** Imput Parameters in PCXMC Dose Calculation Software for Radiation Dose Calculation of Conventional Radiographs ^a,b^

	Small	Medium	Large
**Parameters**			
Height, cm	155	174	195
Weight, kg	45	73	100
Distance between focus and image receptor, cm	124	124	124
Distance between patient exit and image receptor, cm ^a^	15	15	15
**Chest XR, AP**			
Tube voltage, kV peak	125	125	125
Tube current-time product, mAs	0.50	0.50	1
Field of view, cm × cm	43 × 35	43 × 35	43 × 35
**Pelvis XR, AP **			
Tube Voltage, kV peak	70	73	73
Tube current-time product, mAs	20	32	50
Field of view, cm × cm	43 × 35	43 × 35	43 × 35
**Thoracic spines XR, AP **			
Tube voltage, kV peak	70	73	73
Tube current-time product, mAs	12.5	16	32
Field of View, cm × cm	15 × 43	18 × 43	18 × 43
**Thoracic Spine XR, Lat **			
Tube Voltage, kV peak	77	81	85
Tube Current-Time Product, mAs	16	32	50
Field of View, cm × cm	23 × 43	23 × 43	23 × 43
**Lumbar Spines, AP **			
Tube Voltage, kV peak	73	77	81
Tube Current-Time Product, mAs	20	32	50
**Lumbar Spines, Lat **			
Tube Voltage, kV peak	81	85	90
Tube Current-Time Product, mAs	32	50	63
Field of View, cm × cm	18 × 43	20 × 43	20 × 43

^a^ A relatively large distance of 15 cm between patient exit and image was imputed in PCXMC (version 1.5.1, STUK, Radiation and nuclear safety authority, Helsinki, Finland), because trauma patients are usually positioned on top of a spine board.

^b^ Abbreviations: XR, radiography; AP, anterior posterior; Lat, lateral

## Appendix D. Detailed Explanation of Calculation Method for Radiation Exposure (See also Appendix C)

The effective radiation dose of each conventional radiographic investigation was calculated in a phantom study for three representative patient configurations (a patient of 45 kg and 155 cm height, a patient of 73 kg and 174 cm height, and a patient of 100 kg and 195 cm height). Operating parameters for each investigation are displayed in Appendix C. We measured the external air kerma with a semiconductor dosimeter (PTW-Diados, Type 11003-0880, PTW, Freiburg, Germany) that was placed in the X-ray beam at a distance of 124 cm from the focus. We thereafter calculated the dose-area product by taking into account the investigation-specific field of view at the position of the dosimeter. We calculated effective dose (expressed in millisieverts [mSv]) by imputing these measurements, investigation-specific parameters, and patient configuration data into PCXMC dose calculation software (version 1.5.1, STUK Radiation and nuclear safety authority, Helsinki, Finland). This program performed a Monte Carlo simulation and calculated effective doses for all radiographic investigations and for each patient configuration.

## References

[1] Huber-Wagner S, Lefering R, Qvick LM, Körner M, Kay MV, Pfeifer K (2009). Effect of whole-body CT during trauma resuscitation on survival: a retrospective, multicentre study.. The Lancet..

[2] Ruchholtz S, Zintl B, Nast-Kolb D, Waydhas C, Lewan U, Kanz KG (1998). Improvement in the therapy of multiply injured patients by introduction of clinical management guidelines.. Injury..

[3] Demetriades D, Berne TV, Belzberg H, Asensio J, Cornwell E, Dougherty W (1995). The impact of a dedicated trauma program on outcome in severely injured patients.. Arch Surg..

[4] Pehle B, Kuehne CA, Block J, Waydhas C, Taeger G, Nast-Kolb D (2006). [The significance of delayed diagnosis of lesions in multiply traumatised patients. A study of 1,187 shock room patients].. Unfallchirurg..

[5] Self ML, Blake AM, Whitley M, Nadalo L, Dunn E (2003). The benefit of routine thoracic, abdominal, and pelvic computed tomography to evaluate trauma patients with closed head injuries.. Am J Surg..

[6] Brink M, Deunk J, Dekker HM, Kool DR, Edwards MJ, van Vugt AB (2008). Added value of routine chest MDCT after blunt trauma: evaluation of additional findings and impact on patient management.. AJR Am J Roentgenol..

[7] Brenner DJ, Hall EJ (2007). Computed tomography--an increasing source of radiation exposure.. N Engl J Med..

[8] van Vugt R, Dekker HM, Deunk J, van der Vijver RJ, van Vugt AB, Kool DR (2011). Incidental Findings on Routine Thoracoabdominal Computed Tomography in Blunt Trauma Patients.. J Trauma..

[9] Deunk J, Brink M, Dekker HM, Kool DR, van Kuijk C, Blickman JG (2009). Routine versus selective computed tomography of the abdomen, pelvis, and lumbar spine in blunt trauma: a prospective evaluation.. J Trauma..

[10] Brink M, Deunk J, Dekker HM, Edwards MJ, Kool DR, van Vugt AB (2010). Criteria for the selective use of chest computed tomography in blunt trauma patients.. Eur Radiol..

[11] Deunk J, Brink M, Dekker HM, Kool DR, Blickman JG, van Vugt AB (2010). Predictors for the selection of patients for abdominal CT after blunt trauma: a proposal for a diagnostic algorithm.. Ann Surg..

[12] Smits M, Dippel DW, de Haan GG, Dekker HM, Vos PE, Kool DR (2005). External validation of the Canadian CT Head Rule and the New Orleans Criteria for CT scanning in patients with minor head injury.. JAMA..

[13] Oostenbrink J. B., Bouwmans CAM, Koopmanschap MA, Rutten F. (2004). Handleiding voor kostenonderzoek. Methoden en standaard kostprijzen voor economische evaluaties in de gezondheidszorg..

[14] Moore EE, Shackford SR, Pachter HL, McAninch JW, Browner BD, Champion HR (1989). Organ injury scaling: spleen, liver, and kidney.. J Trauma..

[15] Saltzherr TP, Goslings JC (2009). Effect on survival of whole-body CT during trauma resuscitation.. The Lancet..

[16] Fabian TC (2009). Whole-body CT in multiple trauma.. The Lancet..

[17] Oostenbrink JB, Buijs-Van der Woude T, van Agthoven M, Koopmanschap MA, Rutten FF (2003). Unit costs of inpatient hospital days.. Pharmacoeconomics..

[18] Hunink GM, Hunink GM (2001). Decision Making in Health and Medicine with CD-ROM: Integrating Evidence and Values..

